# Sensor Fusion-Based Cooperative Trail Following for Autonomous Multi-Robot System †

**DOI:** 10.3390/s19040823

**Published:** 2019-02-17

**Authors:** Mingyang Geng, Shuqi Liu, Zhaoxia Wu

**Affiliations:** 1 National Key Laboratory of Parallel and Distributed Processing, College of Computer, National University of Defense Technology, Changsha 410073, China; 2 National Key Laboratory of Big Data Management and Analysis, Northeastern University, Shenyang 110000, China; 1701742@stu.neu.edu.cn; 3 School of Control Engineering, Northeastern University at Qinhuangdao, Qinhuangdao 066004, China; 1000429@neuq.edu.cn

**Keywords:** trail following, cooperative perception, multi-robot system, feature fusion

## Abstract

Autonomously following a man-made trail in the wild is a challenging problem for robotic systems. Recently, deep learning-based approaches have cast the trail following problem as an image classification task and have achieved great success in the vision-based trail-following problem. However, the existing research only focuses on the trail-following task with a single-robot system. In contrast, many robotic tasks in reality, such as search and rescue, are conducted by a group of robots. While these robots are grouped to move in the wild, they can cooperate to lead to a more robust performance and perform the trail-following task in a better manner. Concretely, each robot can periodically exchange the vision data with other robots and make decisions based both on its local view and the information from others. This paper proposes a sensor fusion-based cooperative trail-following method, which enables a group of robots to implement the trail-following task by fusing the sensor data of each robot. Our method allows each robot to face the same direction from different altitudes to fuse the vision data feature on the collective level and then take action respectively. Besides, considering the quality of service requirement of the robotic software, our method limits the condition to implementing the sensor data fusion process by using the “threshold” mechanism. Qualitative and quantitative experiments on the real-world dataset have shown that our method can significantly promote the recognition accuracy and lead to a more robust performance compared with the single-robot system.

## 1. Introduction

Autonomously following trails (such as those normally traversed by hikers or mountain-bikers) in the forest is a challenging problem for robotic systems. Trail following is an efficient and safe way for a Micro Aerial Vehicle (MAV) to travel medium and long distances in different environments for various tasks such as search and rescue, environmental mapping, personal videography, and wilderness monitoring. Recently, deep learning technologies have emerged as a powerful tool for various computer vision tasks [1,2,3,4]. DNN and TrailNet [5,6] cast the trail perception problem as an image classification task and enable the system to navigate forest trails more robustly than previous techniques. The trail-following task could be solved by using deep learning models to learn a control strategy that mimics the choice of an expert driver based on the vision data. Unlike the previous works [7,8], which solved trail perception as a segmentation problem (i.e., determining which areas of the input image correspond to the image of the trail), deep learning techniques bypass this need by directly operating on the raw RGB frames and provide high-level information. The accuracy of deep learning technology [5] is comparable to the performance of humans, reaching 85.2%, much higher than that of the traditional method using image saliency (52.3%) [9].

**Figure 1:**
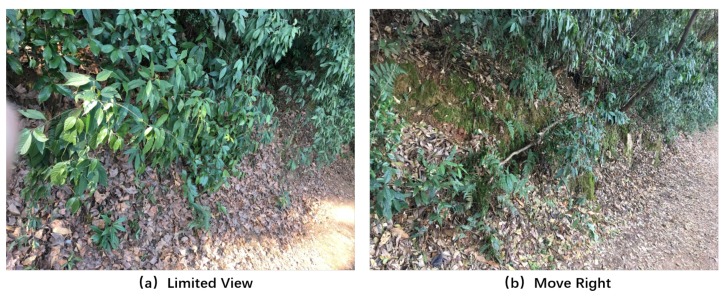
In the single-robot system, when the robot is currently looking sideways (**a**), the trail will not be visible, and the robot cannot deal with this situation. However, from an adjustment instruction of the higher altitude view facing the same direction (**b**), the robot would know it should move right to remain on the trail.

However, existing studies on the trail-following task only concern a single robot [5,6,7,8], which may not lead to a robust performance in some extreme situations. For example, if the robot is currently looking sideways, then the trail is not visible (shown in Figure 1a), which will confuse the robot and impede making decisions. The DNN-based (Deep Neural Network-based) method [5] deals with this problem by performing a two-class classification problem, which could only enable the robot not to go straight without adjustment instructions. Here, we call the situation in Figure 1a “limited view”, which is a great challenge that needs to be solved to enable a robust trail-following performance. Concretely, when there is no trail in the view of the robot, adjustment instructions from other vision data (i.e., move right, shown in Figure 1b) should be given to guide the robot out of the dilemma.

In reality, many robotic tasks, such as search and patrolling, are conducted by a multi-robot system, which can introduce the benefit of robustness from data fusion and information sharing among the robots [10,11,12]. For the trail-following task, the multi-robot system could effectively solve the “limited view” problem by providing the vision data from other robots to help the confused robot. For an illustrative perspective, considering a scene of search-and-rescue after earthquakes, two drones facing the same direction from different altitudes need to climb over the mountain to reach the destination and help to perform the rescue task. The drones could exchange the vision data to complement the vision capability and improve the efficiency for completing the task. Concretely, the high-altitude drone can benefit from the broader view and focus more on global information such as the extension direction of the trail. The low-altitude drone can pay more attention to the details of the trail such as appearance contrast. However, fully exploiting the valuable information from the multi-robot system and deriving a robust fused representation is an extremely challenging task due to the information gap between the high-altitude and low-altitude vision inputs.

Recently, feature fusion technologies have attracted increasing attention in the video categorization domain [13,14,15]. In order to achieve outstanding performance, a great number of works have focused on combining multiple video features and utilizing the inter-class semantic relationships [16,17,18]. The fusion process of multiple features is usually expected to extract the features complementary to each other and derive a robust fused representation. Naturally, motivated by the idea of feature fusion in the video categorization domain, could we apply a similar technology to enable the multi-robot system to share the vision data from different altitudes and perform the trail-following task in a better manner? Concretely, to meet the basic requirements of feature fusion, we simplify the coordination problem by forcing the robots to face towards the same direction and to keep a certain pitching angle. The condition will guarantee that the ground robot is just at the bottom center of the view from the aerial robot, and the decisions for the robots to make are the same. In other words, from the perspective of image recognition, the high-altitude vision input and the low-altitude one have the same label.

In this paper, we apply the feature fusion mechanism to the multi-robot system with the aim of achieving a robust performance on the vision-based trail-following task. We propose the Sensor Fusion-based Cooperative method (SF-Cooper), which contains three feature fusion methods, including SVM (support vector machine), SOFTMAX, and four-layer DNNs (Deep Neural Networks), to generate a discriminative integrated representation effectively from the vision inputs of the robots. The robots are enabled to fuse the features of the vision data from different altitudes on the collective level. By sharing the vision data from different altitudes, the robots could augment their vision and acquire robust adaptability to deal with the “limited view” situations. Besides, considering the Quality of Service (QoS) requirement of the robotic applications, we incorporate the “threshold” mechanism in the system to minimize the latency for the robots to make new decisions and remain coordinated. Concretely, SF-Cooper processes the feature of each robot separately, and the fusion process is implemented only when the maximum probability among the decisions of a certain robot is less than the specified threshold. Qualitative and quantitative experiments are conducted to demonstrate that SF-Cooper can significantly improve the recognition accuracy and lead to a more robust performance compared with the single robot system, especially in the “limited view” situations. Besides, by incorporating the “threshold” mechanism, SF-Cooper can decrease the time latency to a large degree while maintaining the recognition accuracy of the system.

The remainder of this paper is organized as follows. In Section 2, the related work is presented with emphasis on the novelty of our work. Section 3 introduces the problem formulation. Section 4 shows the detailed architecture of SF-Cooper. Section 5 presents the experiments carried out on the testing set and the real-world environment, as well as the analysis of the results. Section 6 provides the link for acquiring our dataset. The discussions of our approach are presented in Section 7.

## 2. Related Work

SF-Cooper covers two research areas, including trail following and feature fusion technologies.

### 2.1. Trail Following

Trail following means making the robots autonomously follow a man-made trail. Especially, for the Micro Aerial Vehicle (MAV) system, the commercial MAVs are mostly teleoperated and do not maintain the ability to follow trails and avoid obstacles autonomously. Nevertheless, sustainable efforts have been made to introduce visual-based approaches to facilitate obstacle avoidance. The technologies can mainly be divided into two kinds: segmentation technologies [7,8] and deep learning approaches [5,6,19]. The segmentation technologies aim to use image saliency [9], based on the assumption that the features of the trail will “stand out” from the other parts of the visual input. The features could be color appearance or intensity [7] contrast with the left and right neighboring regions or the cross-influence between the perception of appearance and the perception of shape [8]. The segmentation-based method is challenging because it needs to find the approximate characteristics first and then highlight [20] them by a series of operations (symmetric, triangular shape [7], or spatial-temporal integration based on virtual ants [8]) to get a satisfying performance.

The trail-following problem could also be cast as an image classification task and solved by deep learning technologies. The earliest one is the DNN research of [5], which defines three kinds of labels (“turn right”, “turn left”, and “go straight”) corresponding to the actions for the robot to remain on the trail. The DNN-based method could bypass the need to choose or design features from the vision data and achieve a better performance than the traditional segmentation-based methods. TrailNet [6] improves upon the DNN-based method by incorporating three additional categories via transfer learning to enable the estimation of both lateral offset and view orientation with respect to the trail. TrailNet also enables a low-level obstacle detection by incorporating an object detection module and a visual odometry component.

However, all the methods mentioned above focus on the trail-following problem for the single-robot system. For the multi-robot system, our previous work [19] accomplished the trail-following task by fusing the decisions of each robot on the collective level. Concretely, we integrate the probabilities (three-dimensional vector) produced by the ground and the aerial robots in order and use the fused representation to perform a final classification. The training process is not end-to-end because we separate the decision extraction process and the decision fusion process. In SF-Cooper, we extract the mid-level features (4096-dimensional vector) from the fully-connected layer of the neural network, which contain more abundant information than the decision, and add a four-layer DNNs feature fusion method to exploit the features fully and derive a more discriminative representation.

### 2.2. Feature Fusion

The feature fusion mechanism is a powerful tool adopted for video classification [21,22]. For example, the visual features, trajectory features, and the audio features can be fused to incorporate more valuable semantic information and achieve a higher classification score [23]. The main feature fusion strategies could be divided into two categories: the early fusion methods and the late fusion methods. Early fusion methods assume that multiple features are explicitly complementary to each other. However, different features may carry task-relevant information at different times; fusing them by naive concatenation may limit the model’s ability to adapt to other situations. Late fusion methods train the models separately and then combine the prediction scores. However, late fusion methods will ignore the feature relationships in the categorization process because the features are processed separately.

Recently, with the development of deep neural networks, a few studies focused on fusing multiple features in neural networks. Concretely, a deep denoised auto-encoder was utilized in [24] to learn a shared representation based on multi-model inputs. Besides, a deep Boltzmann machine was employed in [25] to combine visual and textual features. Similarly, an approach that was based on minimizing the variation of information was proposed to optimize the shared feature learning process. This method can effectively predict the missing input modality according to the available information. In order to achieve dynamic weight adjustments across different models, an attention-based multi-model fusion network [26] was proposed.

In this paper, we apply the feature fusion approaches to the multi-robot system and enable the robots to share the visual inputs for achieving a robust trail-following performance. By forcing the robots to face the same direction and maintain a pitching angle, the feature fusion mechanism can extract valuable information from the vision data of each robot and derive a robust fused representation to perform the classification task. To the best of our knowledge, this is the first paper that applies the feature fusion mechanism to the trail-following task.

## 3. Problem Formulation

The problem we focus on is to make an aerial robot and a ground robot autonomously follow the trails without colliding with obstacles based on the vision inputs only. The trails here are continuous, with no termination, but can be branching to a crossing, which may mislead the group and delay the time to reach the rescue destination. In order to apply the feature fusion mechanism to the coordination process, the two robots are forced to face the same direction and keep a pitching angle of 60°. This condition can guarantee that the ground robot is just at the bottom center of the view from the aerial robot. In other words, from the perspective of image classification, the vision inputs from the two robots should obtain the same label. With the aim of deriving a robust fused representation, the ground and aerial classifiers may focus on different parts to make the extracted information contemplative. Concretely, the aerial classifier should focus more on the global part of the visual input, i.e., the extension direction of the trail. The ground classifier should focus more on the details of the trail, i.e., the appearance comparison.

**Figure 2:**
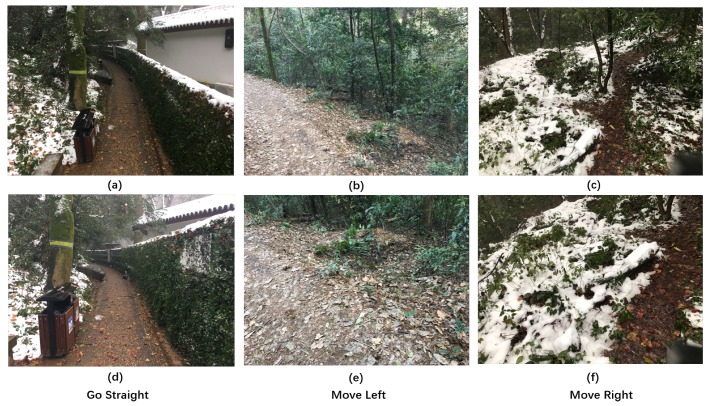
The three labels defined in the problem formulation. The first row represents the aerial vision inputs, and the second row represents the ground visual inputs: (**a**) “Go Straight” label from the high-altitude view; (**b**) “Move Left” label from the high-altitude view; (**c**) “Move Right” label from the high-altitude view; (**d**) “Go Straight” label from the low-altitude view; (**e**) “Move Left” label from the low-altitude view; (**f**) “Move Right” label from the low-altitude view.

In order to complete the trail-following task for the multi-robot system effectively, we need to define decisions that the robots need to make when confronted with a specific situation. From the perspective of image classification, the decision here can be thought of as the label of the corresponding image. As shown in Figure 2, the decisions can be divided into three classes: “move left”, “move right”, and “go straight”. The three classes are “basic” classes that indicate that the robots should move a certain distance towards the given direction. Notably, if the ground robot is faced with the “limited view” problem, the “move left” and “move right” labels are “adjustment” instructions, which can help the ground robot get out of the local dilemma and see the trail again (shown in Figure 1).

**Figure 3:**
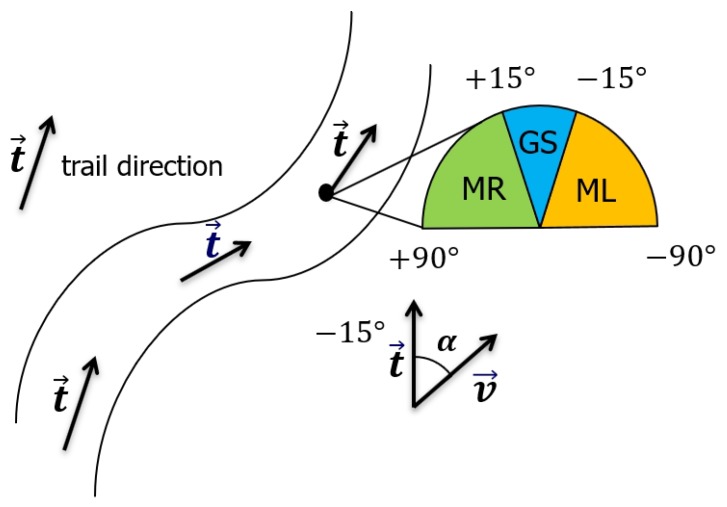
The illustration of the problem formulation. The trail direction represents the direction a hiker would walk with the aim of following the trail. MR, Move Right; GS, Go Straight; ML, Move Left.

The label of a certain vision input is defined as follows. Consider a general scene with a single trail in the wild; our vision input from the ground/aerial robot is an image captured by a camera situated above the ground. Denote $\vec{v}$ as the direction of the camera’s optical axis. We assume that $\vec{v}$ lies on the horizontal plane. Besides, denote $\vec{t}$ as the dominant direction of the trail: we define $\vec{t}$ as the horizontal direction towards which a hiker would start an obstacle-free walk if standing at the position of the robot. Denote α as the signed angle between $\vec{v}$ and $\vec{t}$; the three classes defined above stand for three different situations that the carrier of the camera should implement assuming that the camera is heading in the direction of motion. The details are shown in Figure 3.

- Move Left (ML): if −90° ≤ α<−β; i.e., the trail is heading towards the left part of the image.
- Move Right (MR): if +β≤α < +90°; i.e., the trail is heading towards the right part of the image.
- Go Straight (GS): if −β≤α≤+β; i.e., the trail is heading straight ahead, at least in a close range.

In our experiments, we follow the definition of [5] and consider β = 15°. Here, β stands for the boundary which distinguishes the “go straight” label and the “move left/right” label. In order to derive a robust fused representation of the high-altitude and low-altitude vision inputs, several challenges need to be solved. Firstly, the relevant information has to be extracted from the vision data of each robot in order to express the corresponding responsibility correctly. Then, the feature fusion mechanism needs to make up the drawbacks of the features and make full use of the advantages brought by each robot.

## 4. Method

In this section, we describe the details of our framework SF-Cooper and the three feature fusion methods SVM, SOFTMAX, and four-layer DNNs. SF-Cooper makes a group of mobile robots cooperatively follow the trails autonomously using the sensor data based on the feature fusion mechanism. We will introduce the framework of SF-Cooper, the details of feature fusion algorithms for the multi-robot system, and the theoretical analysis of the performance benefits.

### 4.1. Framework of SF-Cooper

SF-Cooper aims to fuse the sensor data of the robots and derive a robustly-integrated representation for a better classification. The idea behind SF-Cooper is to fuse the extracted features of each vision input and then make the final decision based on the fused representation.

**Figure 4:**
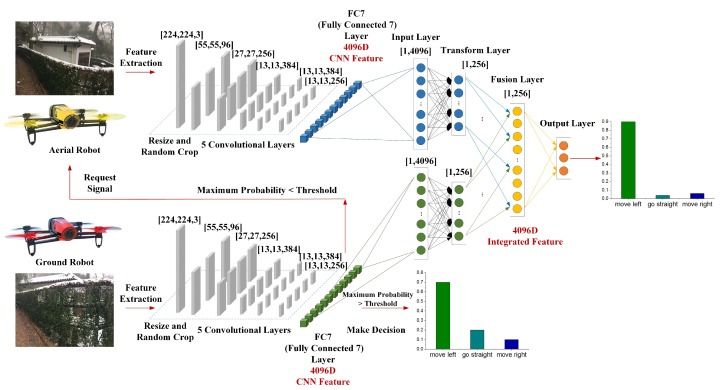
The architecture of the Sensor Fusion-based Cooperative method (SF-Cooper), which is composed of three parts: the input module, the feature extraction module, and the feature fusion module.

The architecture of SF-Cooper is shown in Figure 4. There are two drones in the architecture: the higher one stands for the aerial robot, and the lower one stands for the ground robot. Each drone can capture the images from the corresponding altitude and train the classifier, which will make each robot maintain the ability to extract the features from the visual input and make decisions alone. There are three main responsibilities for the classifiers: extracting features, measuring the time of implementing the feature fusion process, and predicting decisions. The classifier in the upper right corner is the feature fusion module, which is implemented to get a final decision for the two robots based on the integrated representation. The ground and aerial robots first extract the features from the vision inputs. Then, the decision-making process is influenced by the maximum probability among the decisions of the ground robot. The details are illustrated in the next subsection.

### 4.2. Feature Fusion Algorithms for the Multi-Robot System

After the ground robot obtains a visual input, the ground robot will decide whether it is the time to implement the feature fusion process. The ground robot first needs to calculate the maximum probability ψ among the decisions predicted by the ground classifier. Concretely, ψ stands for the maximum value of $\left[PG_{straight},PG_{left},PG_{right}\right]$,which are predicted by the SOFTMAX layer of the ground classifier. Through a comparison between ψ and the specified “threshold”, the ground robot will know whether the feature fusion process is needed now. If the feature fusion requirement is satisfied, the ground robot will first send a classification request signal to the aerial robot to obtain the features from the higher altitude. Then, the aerial robot will implement a recognition process and send the extracted features to the ground robot. Finally, the ground robot will send the low-altitude and high-altitude features to the trained SVM/SOFTMAX/four-layer DNNs modules to get the final decision. If the feature fusion requirement is not satisfied, the ground robot will follow the decision predicted by its own classifier. After the ground robot confirms the decision, the ground robot will send the confirmed decision to the aerial robot to keep coordinated. Algorithm 1 gives the pseudocode of the whole implemented process. Here, “CF” stands for the Classifier.

The feature fusion process aims to integrate the low-altitude and high-altitude features and learn a discriminative shared representation, which can lead to a more robust performance in the vision-based trail-following task. In general, the integrated features output by the feature fusion algorithms should be more discriminative than both the low-altitude features and the high-altitude features. Three feature fusion algorithms are used in SF-Cooper. We first extract the low-altitude and the high-altitude features from the trained ground and aerial classifiers, respectively. Then, the extracted features will be sent to the feature fusion modules. For the SVM/SOFTMAX classifier, we integrate the low-altitude and the high-altitude features in order. Then, the integrated vector after splicing and the corresponding label are used as the input for training.

**Algorithm 1** The feature fusion algorithm using SVM/SOFTMAX/four-layer DNNs.**Input:** Image $X_{ground}$ acquired by the ground robot, image $X_{aerial}$ obtained by the aerial robot, the trained ground and aerial classifiers to extract the features from the vision input, and the trained feature fusion modules SVM/SOFTMAX/four-layer DNNs to complete the feature fusion process.**Output:** Class label *C* defined in the problem formulation, which is then transferred as the flight command of the robot.  1:Initialize and take off from the ground and aerial drones (robots).  2:The ground robot gets the vision input.  3:$C_{ground}$ = $CF_{ground}\left(X_{ground}\right)$  4:The ground robot gets the list $\left(PG_{straight},PG_{left},PG_{right}\right)$ from the SOFTMAX layer of the trained ground classifier.  5:$\psi =max(PG_{straight},PG_{left},PG_{right})$
  6:**if** ψ<Threshold **then**  7:    The ground robot extracts the low-altitude features $f_{ground}$ using the trained ground classifier from the low-altitude vision input and sends a classification request signal to the aerial robot.  8:    The aerial robot extracts the high-altitude feature $f_{aerial}$ using the trained aerial classifier from the high-altitude vision input and sends the features to the ground robot.  9:    The ground robot sends the low-altitude and the high-altitude features to the SVM/SOFTMAX/four-layer DNNs modules to implement the feature fusion process. 10:    The ground robot gets the final decision based on the integrated representation from the feature fusion modules. $C=C_{SVM/SOFTMAX/4-layerDNNs}$ 11:**else** 12:    $C=C_{ground}$ 13:**end if** 14:Both the aerial robot and the ground robot take the *C* command.

For the four-layer DNNs, as demonstrated in Figure 4, the model contains four layers: the input layer, transformation layer, fusion layer, and output layer. Both the low-altitude and the high-altitude features are used as the input of the first layer. The inputs are then transformed using a hidden layer with 256 neurons for each kind of feature, respectively. The transformed features are then fused with a fusion layer containing 256 neurons. Finally, the fused features are converted to classification scores through the last layer. Notably, experiments [23] have shown that four layers are empirically found to be suitable for the feature fusion process. From an illustrative perspective, the transition equation for the fusion layer can be written as follows:

(1) $$Z_{F}=\sigma \left(W_{E}^{l}Z_{E}^{l}+W_{E}^{h}Z_{E}^{h}+b_{E}\right)$$

Here, *E* represents the index of the last layer of feature transformation, and *F* represents the index of the fusion layer (i.e., F=E+1). Therefore, $Z_{E}^{l}$ and $Z_{E}^{h}$ represent the extracted mid-level representation for the low-altitude and high-altitude features respectively. As demonstrated in Equation (1), the mid-level representation is firstly linearly transformed by the weight matrix $W_{E}^{l}$/$W_{E}^{h}$ and then non-linearly mapped to generate the integrated representation $Z_{F}$ using a sigmoid function σ. In order to perform the feature fusion process by exploring correlations and diversities simultaneously, we can minimize the following cost function to learn the optimal weights for each layer in the four-layer DNNs,

(2) $$min_{W}\sum_{i=1}^{N}l\left(y_{i}^{\prime },y_{i}\right)+\frac{\lambda }{2}(\sum_{p=1}^{E}(∥W_{p}^{l}{∥}_{F}^{2}+∥W_{p}^{h}{∥}_{F}^{2})+\sum_{p=F}^{P-1}∥W_{p}{∥}_{F}^{2}),$$

where the function $\sum_{i=1}^{N}l\left(y_{i}^{\prime },y_{i}\right)$ is the empirical loss on the training data, which summarizes the discrepancy between the outputs of the network $y_{i}^{\prime }$ and the ground-truth labels $y_{i}$. *N* denotes the number of classes. The remaining part is a regularization term preventing overfitting. *P* stands for the total number of layers in the four-layer DNNs, and λ balances the contribution of the regularization term for the whole loss function.

### 4.3. Performance Benefits of SF-Cooper

The benefits of introducing the feature fusion mechanism into the trail following problem can be analyzed theoretically from two aspects.

#### 4.3.1. Compared with the Single-Robot Method

We demonstrate the superiority of the accuracy benefits of feature fusion mechanism compared with the single ground robot system. By introducing the feature fusion mechanism into the trail-following task, the cognitive capability of the ground robot can be improved. Generally, the final recognition accuracy of all unrecognized samples can be increased from 0 to (1−p)F, where *F* is the recognition accuracy of the SVM/SOFTMAX/four-layer DNNs feature fusion modules, and *p* is the false positive rate of the measurement mechanism on feature fusion time (i.e., the ground classifier recognizes the image incorrectly, but the feature fusion requirement is not satisfied). However, the promotion of recognition accuracy is not evident for all types of samples. The instability of the measurement mechanism on feature fusion time should be considered. For example, a sample that has already been correctly recognized by the ground robot may be mistakenly sent to implement the feature fusion process. Therefore, we will provide detailed analyses of the accuracy benefits.

Considering the impact of the measurement mechanism on feature fusion time, the accuracy promotion of SF-Cooper can be calculated in the following method. We denote *G* as the recognition accuracy of the ground classifier. Then, the total accuracy *H* can be calculated as follows:

(3) H=G(1−p)+GpF+(1−G)(1−p)F.

The explanation of the symbols in Equation (3) is shown in Table 1. Here, G(1−p) refers to the samples that are classified correctly by the ground robot and directly taken as the final decision (the feature fusion requirement is not satisfied). GpF stands for the samples that are already classified by the ground robot, but mistakenly sent to deal with further and finally classified correctly by the feature fusion algorithms. (1−G)(1−p)F refers to the samples that are wrongly classified by the ground robot, but finally rectified, attributed to the correctness of the measurement mechanism on feature fusion time and the feature fusion algorithms. Through a simple deformation, we could obtain the following theorem:

**Theorem** **1.** *Recognition accuracy H≥G if and only if $F\geq \frac{Gp}{Gp+(1-G)(1-p)}$.*

With the help of the aerial classifier and the feature fusion algorithms, a large proportion of unrecognized samples can be corrected from a statistical point of view, and the accuracy promotion would be greater with more uncertainty in the environment, i.e., the samples in the “limited view” situations.

#### 4.3.2. Comparison without the Measurement Mechanism on Feature Fusion Time

The objective of introducing the measurement mechanism on feature fusion time is to satisfy the QoS requirement of the robotic applications, particularly the latency on the samples where the feature fusion process is not necessary. The total latency of SF-Cooper $l_{s}$ can be calculated by:

(4) $$l_{s}=l_{g}+\mu l_{f},$$

where $l_{g}$ is the average latency of the ground classifier, $l_{f}$ is the latency of the feature fusion modules, and μ is the probability that the feature fusion time measurement mechanism believes it is time to implement the feature fusion process. The explanation of the symbols in Equation (4) is shown in Table 2. Compared with the method that the feature fusion process is implemented all the time, we can obtain the following theorem:

**Theorem** **2.** If $\mu \leq 1-\frac{l_{g}}{l_{f}}$, then $l_{s}\leq l_{f}$.

$l_{g}$ is significantly less than $l_{f}$ in practice because the transmission of the aerial features costs much time. As a reference, $\frac{l_{g}}{l_{f}}$ in the experiments presented in the next section is frequently below 0.1. Consequently, $1-\frac{l_{g}}{l_{f}}$ is usually a number near one, and the final average latency for the classification is certainly less than the method without the measurement mechanism on feature fusion time.

## 5. Experiments

In this section, we will introduce our experiments from the following three aspects: the datasets, the details of feature extraction and fusion, the experimental results, and discussions.

### 5.1. Datasets

The dataset is composed of two parts: the “basic” set and the “limited view” set. The “basic” set is acquired as follows: we make two Parrot Bebop Drones [27] coordinated to follow the trail using the “move left”, “move right”, and “go straight” commands. Concretely, the low-altitude drone flies at an altitude of 1.7 m, which stands for the view of a medium ground robot. The high-altitude drone flies at an altitude between 3.2 and 4.7 m (affected by the realistic conditions such as branches) to guarantee that the aerial robot just has a global view near the ground robot. We make the two drones face the same direction and follow each trail three times (one pointing 30° to the left, one pointing straight ahead, and one pointing 30° to the right) to collect the dataset. Images and commands are exchanged via a WiFi connection between our telephones and the Bebop drones. From the Bebop’s stream connection, we receive an image of a resolution of 1920 × 1080 pixels. The dataset covers approximately 5 km of hiking trails acquired at altitudes ranging from 300–900 m for different times of the day and weather. Meanwhile, many different trail types and surroundings are represented, ranging from sloped narrow alpine paths to wider forest roads.

Each pair of images is labeled by an expert driver, associated with its ground truth class. We also augment the training dataset by synthesizing left/right mirrored versions of each training image. In detail, a mirrored training image of class “move right/left” yields a new training sample for class “move left/right”. A mirrored “go straight” training sample yields another “go straight” training sample. Additionally, mild affine distortions (±10% translation, ±15% rotation, ±10% scaling) are applied to the training set with the aim of increasing the number of samples further. To train a classifier that is robust to noise, we generate additional images and augment our dataset size by adding Gaussian white noise of mean 0 and variance 0.01 to our dataset. We also collect the “limited view” samples by adjusting the facing direction of the ground robot from 50 locations (35 for train and 15 for test) and 80 pairs on each. The two “adjustment” instructions for the aerial dataset are represented as averages. Notably, the labels of the “limited view” samples for the ground robot are given by the corresponding instruction of the aerial robot. Besides, all the samples have been split into disjoint training (15,120 pairs) and testing (4808 pairs) sets. The split is implemented by carefully avoiding that the same scene appears in both the training and testing set, and the classes are evenly represented within each set.

### 5.2. Feature Extraction and Fusion

For the feature extraction modules, we train the ground and aerial classifiers to extract the represented features using the low-altitude and the high-altitude datasets. We start with the AlexNet model, which is composed of five convolutional layers and three fully-connected layers. Due to the reason that the dataset contains three labels, we replace the last fully-connected layer $FC_{8}$ with a layer composed of three nodes. In the feature extraction process, the images in all three datasets are first re-scaled to 224 × 224-pixel images, and the network is trained with back-propagation for 90 epochs, which requires 60 h on a workstation equipped with the Nvidia Tesla K80 GPU and Nvidia Cudnn (DELL, Shenzhen, China) [28]. The learning rate of the ground and aerial classifiers is initially set to 0.005, then scaled by a factor of 0.95 per epoch. We extract a 4096-dimensional feature representation from $FC_{7}$, which is the output of the seventh fully-connected layer. We disable the “mirror” operation of the data augmentation process during the process of training and testing because this operation does not suit our experiment: an image with the “move right” command will become an image for which a robot should move to the left by mirroring, but the flipped image still has the label of “move right”.

The feature fusion module and the feature extracting modules are not trained end-to-end. For the four-layer DNNs feature fusion module, we follow the experimental settings in [23] by setting the learning rate to 0.7 and fixing $\lambda_{1}$ to a small value of 3 × 10^−5^ with the aim of preventing overfitting. The training for the feature fusion module will stop if it reaches the maximal epochs (we set 300 here) or the training error stops to decrease (with difference less than 1 × 10^−5^) in the last ten epochs.

### 5.3. Results and Discussion

In this subsection, we test the performance of SF-Cooper for two methods. The first one is to use the testing dataset with the aim of showing the accuracy benefits compared with the single-robot system, particularly on the “limited view” testing set. The second one is to implement SF-Cooper on a real platform with the aim of showing the effectiveness of our approach.

#### 5.3.1. Experiments on the Testing Dataset

We divide the testing set into two parts: the “basic” set and the “limited view” set. Firstly, we tested the ground and aerial classifiers and the feature fusion algorithms on the “basic” testing set. The threshold here was set to one. In order to measure the advantages of the three feature fusion algorithms, we also made a comparison with our previous work [19] by implementing a series of experiments, which fused the decisions of the aerial and ground robots. Notably, the decision here refers to the tuple $\left[P_{straight},P_{left},P_{right}\right]$, which was predicted by the SOFTMAX layer of the aerial/ground classifier. The difference between the three-dimensional decision and the 4096-dimensional feature was that the feature contained more information than the decision, but needed more time to transmit.

As shown in Table 3, the accuracy of the aerial classifier (78.4%) was a little better than that of the ground classifier (77.5%), which can be attributed to the broader view (a clear view of the extension direction) of the aerial robot. For the feature fusion algorithms, the four-layer DNNs method achieved the highest accuracy of 95.2%, which validates the superiority of the integrated representation learned by the fusion layer. Additionally, the SVM and SOFTMAX feature fusion algorithms also obtained satisfying accuracies, of 89.5% and 88.7%, respectively. For the decision fusion methods, the SVM method achieved the best performance of 92.6%, which was 2.2% better than the accuracy of the four-layer DNNs. Therefore, we conclude that the four-layer DNNs method is more suitable for feature fusion, and the SVM method is more suitable for decision fusion. The reason is that the four-layer DNNs model needs higher dimensional features to extract valuable information, while the SVM classifier may be capable of generating an accurate hyper-plane for classifying the low-dimensional features (six-dimensional fused decision). Besides, we can see that all the feature fusion algorithms outperformed the single-robot system (the ground robot only and the aerial robot only), which indicates that the feature fusion modules managed to extract valuable information and make the integrated representation more discriminative.

Furthermore, we link back to Theorem 1 to show how the theorem is with respect to the experimentally-obtained results. For Theorem 1, experiments have shown that the accuracy of the ground classifier *G* was 0.775, and the accuracy of the feature fusion module *F* was 0.952 (when the threshold was set to one). Therefore, Theorem 1 can be transformed as: H≥G if and only if p≤0.852. This means that in order to make SF-Cooper outperform the ground classifier, the false positive rate of the measurement mechanism on feature fusion time only needs to be less than 0.852, which is an extremely elementary standard to satisfy and indicates the effectiveness of SF-Cooper.

**Figure 5:**
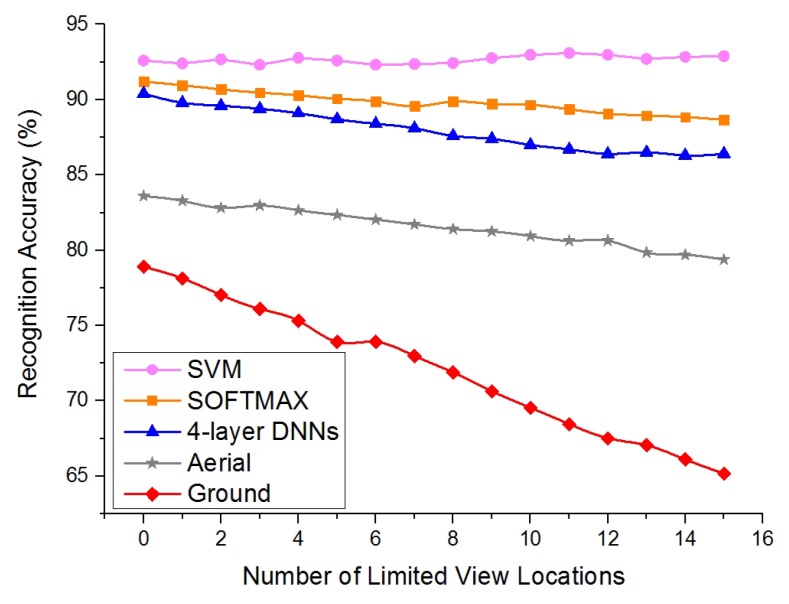
The performance of decision fusion methods with the “limited view” samples introduced to the system.

Considering the problem of the “limited view”, we used the “limited view” testing set and added the corresponding samples from 15 untrained locations to the “basic” testing set gradually. The threshold here was set to one, and Figure 5 illustrates the performance comparison between the decision fusion methods. As shown in Figure 5, as more samples from the locations of limited view were added, the performance of the aerial classifier was relatively more stable than the ground classifier, which can be attributed to the broader view. The accuracy of the ground classifier declined drastically (from 79.2% without limited view locations to 65.3% with 15 locations). The performance of SF-Cooper using the “SVM” method did the best job. It was considerably more stable (accuracy is 92.6% without limited view locations and 92.8% with 15 locations tested). Therefore, compared with the ground robot system, we gain an accuracy promotion of 27.5% at a maximum, and this accuracy promotion is expected to become larger when more limited view samples are added into the system. The performance of the “SOFTMAX” method was also optimistic, just a little lower than that of “SVM”. The outperforming of the “SVM” method over the “SOFTMAX” method can be explained due to the fact that the SVM classifier can extract more valuable information from the integrated features. It is interesting to find that the feature fusion modules can even recognize and rectify the situations where both the aerial and ground classifiers predict wrong decisions. This will help to explain why the feature fusion algorithms show a more stable performance than the aerial classifier. Therefore, the features extracted by the ground and aerial classifiers maintain their diversities and can be supplemented with each other.

**Figure 6:**
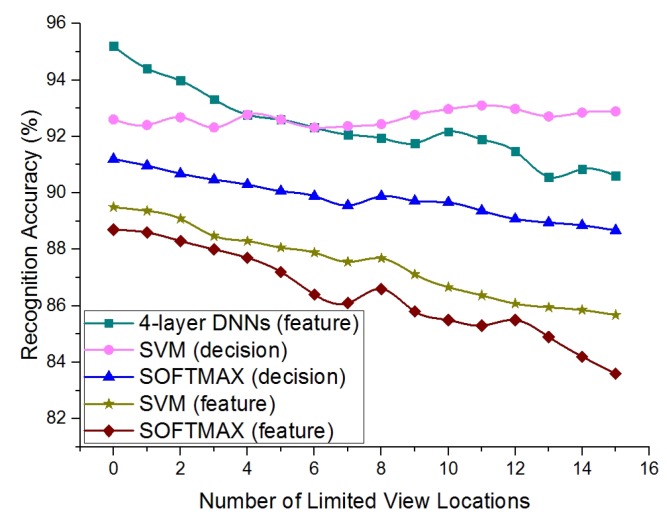
The comparison between the feature fusion methods and the decision fusion methods with the “limited view” samples introduced to the system.

We further implemented the comparison experiments by exploring the feature fusion methods of SVM/SOFTMAX/four-layer DNNs. As shown in Figure 6, the four-layer DNNs method with feature fusion did not have a stable performance, and declined from 95.2% without limited view locations to 90.7% with 15 locations. The reason for the instability may because the low-altitude features contained much useless information and misled the final decision of the feature fusion module. Instead, the SVM method with decision fusion successfully balanced the useless low-altitude decision and the valuable high-altitude decision and showed a more stable performance. Table 4 and Table 5 record the confusion matrix of the SVM method with the decision fusion on the “basic” set and “limited view” set. Table 6 and Table 7 record the confusion matrix of the four-layer DNNs method with feature fusion on the “basic” set and “limited view” set. We can see that the accuracy of the feature fusion method on the “basic” test set was higher than that of the decision fusion method. However, on the “limited view” set, the feature fusion method did not perform better than the decision fusion method. Therefore, we conclude that the feature fusion method is more suitable for the “basic” set and that the decision fusion method is more suitable for the “limited view” problem.

**Figure 7:**
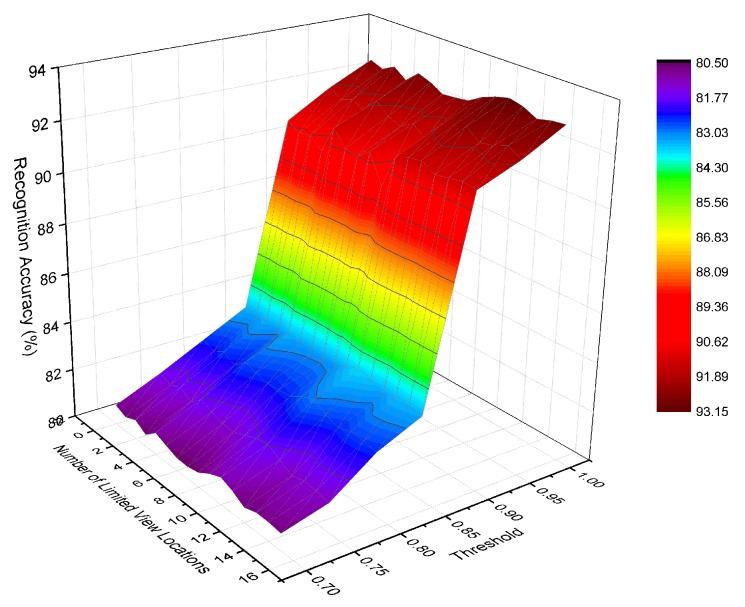
Results of SVM classifier with different combinations of variables.

Considering the effect of the measurement mechanism on feature fusion time, we also implemented a series of experiments using the SVM method with decision fusion whose independent variables are related to SF-Cooper: the number of limited view locations in the testing set and the threshold. Figure 7 presents the recognition accuracy with different combinations of the two variables. Regardless of the number of limited view locations, the increase in accuracy was minimal as the threshold increased from 0.70–0.85, but the accuracy increased sharply when the threshold changed from 0.85–0.90. Therefore, we can conclude that the maximum probability among the decisions of the ground classifier was always larger than 0.85 when it gave a wrongly-classified result. More limited view locations correspond to higher accuracy promotion when the threshold was changed from 0.90–1 because the accuracy declined at a lower speed. Therefore, the inter-regional [0.90, 1] was approximate for the value of threshold, which can be further dealt with based on the requirement of the realistic system.

**Figure 8:**
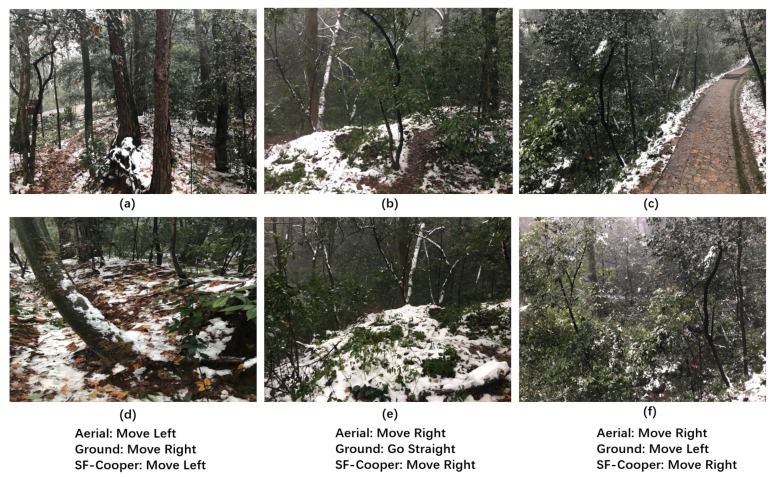
Three “limited view" samples of the ground robot facing an inappropriate direction. The final decisions were successfully recognized by the feature fusion modules with the help of the high-altitude features: (**a**) the high-altitude “Move Left” labeled sample; (**b**) the high-altitude “Move Right” labeled sample; (**c**) the high-altitude “Move Right” labeled sample; (**d**) the low-altitude “limited view” sample corresponding to (**a**); (**e**) the low-altitude “limited view” sample corresponding to (**b**); (**f**) the low-altitude “limited view” sample corresponding to (**c**).

**Figure 9:**
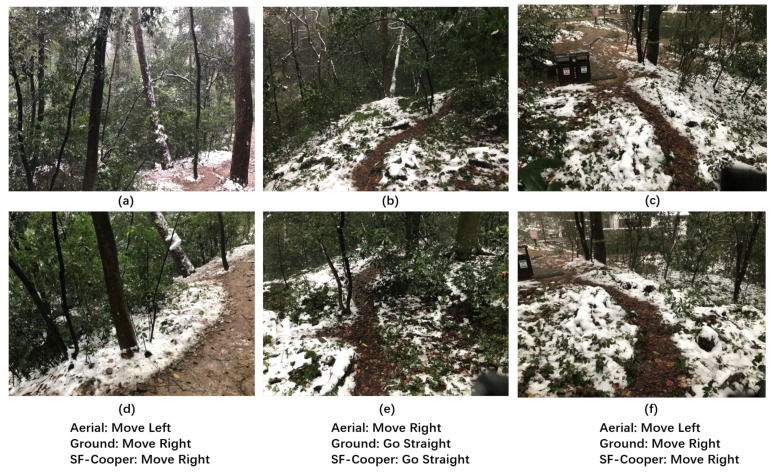
The samples for which the aerial robot predicted the wrong decision, but which was finally rectified by SF-Cooper: (**a**) the high-altitude “Move Right” labeled sample; (**b**) the high-altitude “Go Straight” labeled sample; (**c**) the high-altitude “Move Right” labeled sample; (**d**) the low-altitude “Move Right” labeled sample corresponding to (**a**); (**e**) the low-altitude “Go Straight” labeled sample corresponding to (**b**); (**f**) the low-altitude “Move Right” labeled sample corresponding to (**c**).

**Figure 10:**
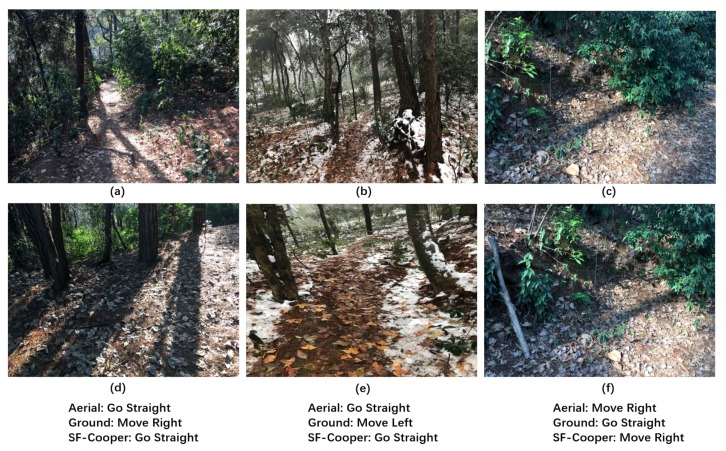
The samples for which the ground robot predicted the wrong decision, but was finally rectified by SF-Cooper: (**a**) the high-altitude “Go Straight” labeled sample; (**b**) the high-altitude “Go Straight” labeled sample; (**c**) the high-altitude “Move Right” labeled sample; (**d**) the low-altitude “Go Straight” labeled sample corresponding to (**a**); (**e**) the low-altitude “Go Straight” labeled sample corresponding to (**b**); (**f**) the low-altitude “Move Right” labeled sample corresponding to (**c**).

We further report the cases in which the ground robot and the aerial robot have disagreements on the decision. Figure 8 illustrates three “limited view” samples, which were finally correctly recognized by the feature fusion modules. We observe that the instances were caused by an inappropriate facing direction of the ground robot. After the ground robot takes the correct command predicted by SF-Cooper, the ground robot will see the trail again and continue to perform the task. Figure 9 illustrates the samples, in which the aerial robot predicted the wrong decision, but which was finally rectified by SF-Cooper. We observe that the aerial robot was often misled by the extension direction of the trail, i.e., in Figure 9b,e, the trail extended to the right part of the image, but from the current view, the robot needed to go straight to remain on the trail. Figure 10 shows the samples, in which the ground robot predicted the wrong decision, but finally rectified by SF-Cooper. We observe that the ground robot easily made mistakes when there were some confusing surroundings like shadows and slight slopes. For example, in Figure 10c,f, the ground robot was confused by the shadow of the trees and predicted a decision of “go straight”; however, there was a corner in front, and the robot needed to “move right” to get out of the dilemma.

#### 5.3.2. Implementation on a Real Platform

We implemented the whole architecture on a real platform using the classifiers that were trained by the low-altitude and the high-altitude datasets. Inexpensive components were chosen by us with the aim of ensuring a robust and flexible platform for forest flight experiments. The quadcopter we chose is the Parrot Bebop Drone, which contains an ultrasound sensor for measuring ground altitude, an onboard computer, and a single forward-facing camera. For each Bebop, commands and images are exchanged via a WiFi connection between the corresponding host machine. The WiFi connection covers a signal range of 250 m. The image captured by each Bebop was 1920 × 1080 pixels. Each classifier was run on the corresponding host machine 2015 Alienware 15 (NVIDIA GeForce GTX 970M, Intel Core i5, 16 GB memory, (DELL, Kunshan, China)), running Ubuntu 16.04.

The testing scenario was the same as that where we collected the dataset. The only difference was that the two parrot bebop drones were not controlled by humans, but directly connected to a DELL R430 server via WiFi. We implemented a simple reactive controller, which translated the output of SF-Cooper (using the SOFTMAX method) to control signals as follows. Yaw (i.e., steering) is proportional to P(MR)−P(ML); a positive value steers the robot to the right, and a negative value steers the robot to the left. Speed is proportional to P(GS). The two drones take the control signals from the corresponding output.

**Figure 11:**
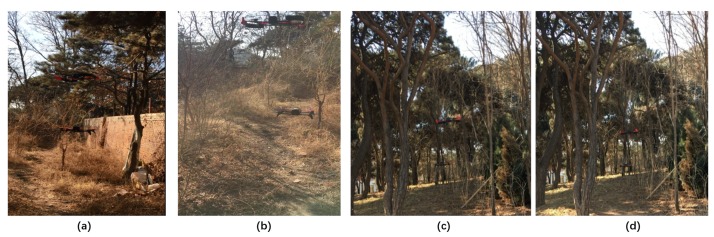
Four scenes in our real-world experiments: (**a**) experiments in scenario 1; (**b**) experiments in scenario 2; (**c**) experiments in scenario 3; (**d**) experiments in scenario 4.

**Figure 12:**
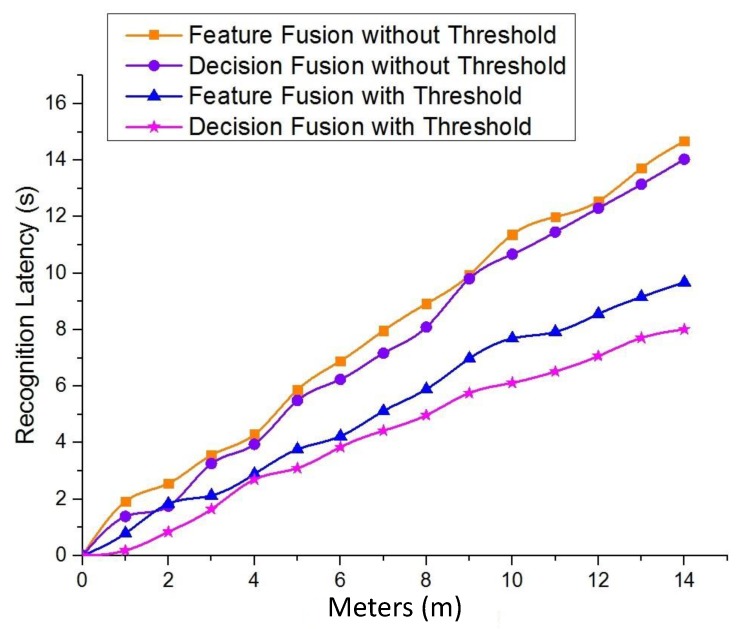
Recognition latency comparison between the decision/feature fusion methods with and without the “threshold” mechanism.

We tested SF-Cooper by autonomously navigating in several different environments, including a 300-m zigzag forest trail with four turns, a 100-m straight trail, and a 500-m zigzag trail over hilly terrain. The trail over hilly terrain was extremely difficult to detect due to the difficulty of defining features contrasted with the surrounding grass. The two drones were able to follow the trails successfully in the above environments. Some scenes in our experiments are illustrated in Figure 11. In order to show our optimization of the time delay compared with the method without the measurement mechanism on feature fusion time, we repeated the experiments with the threshold of one and recorded the recognition latency among the four methods: feature fusion without threshold, decision fusion without threshold, feature fusion with threshold, decision fusion with threshold. The variable here is meters, and one meter means one decision-making process. From Figure 12, we can see that the feature fusion methods required longer latency than the decision fusion methods. Additionally, the response time of the methods without the “threshold” mechanism maintained a satisfying QoS, which will guarantee that the two drones can deal with the emergencies without colliding with obstacles and successfully follow the trails for a few hundred meters. Besides, we found that the latency of the ground classifier $l_{g}$ was about 0.18 and that the latency of the feature fusion module was about 1.92. Therefore, for Theorem 2, $\frac{l_{g}}{l_{f}}$ was frequently below 0.1, and the probability to implement the feature fusion process μ was certainly less than $1-\frac{l_{g}}{l_{f}}$ (a value near one). This further demonstrates that the latency of SF-Cooper was definitely less than the method without the measurement mechanism on feature fusion time.

## 6. Materials and Methods

This can refer to the data set in Data 1 (Supplementary Material).

## 7. Conclusions

This paper presented an approach that applied feature fusion technologies to the multi-robot system with the aim of realizing autonomous trail following. The proposed architecture SF-Cooper provided a convenient way to transfer and update in the future. By fusing the visual features of the ground robot and the aerial robot, the trail-following task was accomplished effectively. The test results in the real-world environment showed that our method could achieve great accuracy improvement with a satisfying latency and had successfully dealt with the “limited view” problem produced by the single-robot system. Concretely, the SVM classifier with the decision fusion module was more suitable for the “limited view” situations. The four-layer DNNs classifier with the feature fusion module could better solve the “basic” situations.

However, several accessible aspects should be considered in the future to improve it. More robots should be introduced into our system in order to realize the accuracy optimization from multiple altitude views. The whole framework could be implemented to train end-to-end, which means that there should be no separated classifiers. Furthermore, we will extend the target space from discrete space to continuous space.

## Figures and Tables

**Table 1:** Illustration of the symbols in Equation (3).

Symbol	Description
*G*	the recognition accuracy of the ground classifier
*p*	the false positive rate of the feature fusion time measurement mechanism
*F*	the recognition accuracy of the feature fusion module
*H*	the total accuracy of SF-Cooper

**Table 2:** Illustration of the symbols in Equation (4).

Symbol	Description
$l_{s}$	the recognition latency of SF-Cooper
$l_{g}$	the recognition latency of the ground classifier
μ	the probability to implement the feature fusion process
$l_{f}$	the recognition latency of the feature fusion module

**Table 3:** The results on the “basic” testing set averaged over 5 runs (%).

Fusion Method	Ground	Aerial	SVM	SOFTMAX	4-Layer DNNs
decision	77.5 ± 0.93	78.4 ± 1.46	92.6 ± 0.48	91.2 ± 0.73	90.4 ± 1.33
feature	–	–	89.5 ± 0.67	88.7 ± 0.98	95.2 ± 1.05

**Table 4:** Confusion matrix of SVM on the “basic” set using the decision fusion method averaged over 5 runs.

Label	ML	MR	GS	Total
ML	1114	21	63	1198
MR	10	1086	92	1188
GS	45	38	1139	1222
Total	1169	1145	1294	3608

**Table 5:** Confusion matrix of SVM on the “limited view” set using the decision fusion method averaged over 5 runs.

Label	ML	MR	GS	Total
ML	555	37	8	600
MR	20	569	11	600
GS	–	–	–	–
Total	575	606	19	1200

**Table 6:** Confusion matrix of 4-layer DNNs on the “basic” set using the feature fusion method averaged over 5 runs.

Label	ML	MR	GS	Total
ML	1179	13	6	1198
MR	22	1113	53	1188
GS	43	36	1143	1222
Total	1244	1162	1202	3608

**Table 7:** Confusion matrix of 4-layer DNNs on the “limited view” set using the feature fusion method averaged over 5 runs.

Label	ML	MR	GS	Total
ML	457	58	85	600
MR	78	471	51	600
GS	–	–	–	–
Total	605	569	26	1200

## Supplementary Materials

Data 1 is available online at https://pan.baidu.com/s/1o-bkke3XVIpyrR9yYgUXaA.
